# Family Planning, Fertility, and Career Decisions Among Female Oncologists

**DOI:** 10.1001/jamanetworkopen.2022.37558

**Published:** 2022-10-31

**Authors:** Anna Lee, Aleksandra Kuczmarska-Haas, Shraddha M. Dalwadi, Erin F. Gillespie, Michelle S. Ludwig, Emma B. Holliday, Fumiko Chino

**Affiliations:** 1 Department of Radiation Oncology, University of Texas MD Anderson Cancer Center, Houston; 2 Department of Human Oncology, University of Wisconsin School of Medicine and Public Health, Madison; 3 Department of Radiation Oncology, University of Texas Health Science Center, MD Anderson Mays Cancer Center, San Antonio; 4 Department of Radiation Oncology, Memorial Sloan Kettering Cancer Center, New York, New York; 5 Department of Radiation Oncology, Baylor College of Medicine, Houston, Texas

## Abstract

**Importance:**

Female oncologists often spend their childbearing years in training and establishing careers, with many later experiencing fertility issues when starting a family. Physician fertility and family planning are rarely discussed during training. Attitudes among female oncologists regarding family planning are unknown.

**Objectives:**

To understand barriers to family planning as well as the association of fertility treatment with career decisions and to assess experiences of pregnancy-based discrimination among female oncologists.

**Design, Setting, and Participants:**

In this survey study, a novel 39-item questionnaire was distributed to US female oncologists from May 7 to June 30, 2020, via email and social media channels. Questions regarding factors associated with family planning, maternity leave, and discrimination were included.

**Main Outcomes and Measures:**

The distribution of survey responses was compared by oncology subspecialty. Multivariable logistic regression was performed to determine independent variables for discrimination experienced during maternity leave.

**Results:**

Responses were collected from 1004 female oncologists. Most respondents (847 [84.4%]) were married, and 713 (71.0%) were currently working full-time. A total of 351 oncologists (35.0%) worked in radiation oncology, 344 (34.3%) in medical oncology, 186 (18.4%) in surgical oncology, and 91 (9.1%) in pediatric oncology. A total of 768 respondents (76.5%) had children, and of these, 415 (41.3%) first gave birth during postgraduate training, and 275 (27.4%) gave birth in years 1 to 5 as an attending physician. Almost all respondents (951 [94.7%]) stated that their career plans were at least somewhat associated with the timing of when to start a family. Having a supportive partner was the most commonly cited positive association with family planning (802 [79.9%]), while long work hours and heavy workload (669 [66.6%]) were the most common negative factors. One-third (318 [31.7%]) had miscarried, and 315 (31.4%) reported difficulty with infertility that required fertility counseling and/or treatment; 660 (65.7%) thought fertility preservation should be discussed with women during medical school and/or residency. One-third (312 [31.1%]) reported experiencing discrimination during pregnancy, and 332 (33.1%) stated they experienced discrimination for taking maternity leave. On multivariable logistic regression, having more than 1 child was associated with increased likelihood of experiencing discrimination during maternity leave (2 children: odds ratio, 1.62 [95% CI, 1.10-2.39]; *P* = .02; ≥3 children: odds ratio, 1.84 [95% CI, 1.14-2.95; *P* = .01).

**Conclusions and Relevance:**

In this survey study of female oncologists, 1 in 3 reported experiencing infertility and 1 in 3 stated they experienced discrimination during pregnancy and/or for taking maternity leave. Systemic changes are necessary to ensure women are supported and able to advance equitably in the field.

## Key Points

**Question:**

What are the barriers to family planning and the association of fertility concerns with career decisions among female oncologists?

**Findings:**

In this survey study including 1004 female oncologists, 95% stated that their career plans were at least somewhat associated with the timing of family planning; 31% reported difficulty with infertility, requiring counseling or treatment; and up to 33% reported discrimination during pregnancy and maternity leave.

**Meaning:**

This study suggests that female oncologists face difficult career choices and discriminatory practices when attempting to build a family; systemic changes are needed to ensure that women are supported and able to advance equitably within oncology.

## Teaser

This survey study examines barriers to family planning as well as the association of fertility treatment with career decisions and assesses pregnancy-based discrimination among female oncologists.

## Introduction

Female physicians often spend their childbearing years in training and establishing careers. In the US, as many as one-fourth of female physicians experience infertility,^1^ more than twice the rate of the general population, according to the Centers for Disease Control and Prevention.^2^ This is a substantial problem, as infertility has physical, emotional, and financial consequences^3,4,5^ and may have a negative association with physician well-being.^6^ Currently, physician fertility and family planning are rarely discussed or taught in medical school or postgraduate training.

A survey study of physician fertility found that more than half have attempted to conceive earlier and that 16.7% of those who received a diagnosis of infertility stated they would have used cryopreservation to preserve fertility.^1^ Another study found that women in academics who delay childbearing for professional advancement may end up “involuntarily childless” owing to underestimations of infertility concerns.^7,8^ On average, female physicians may have their first child 7.4 years later than the general population,^1^ at which point they are already considered advanced maternal age (defined as ≥35 years at the time of giving birth).

Oncology consists of various surgical and nonsurgical subspecialties, representing a microcosm of the landscape of women in medicine; training often requires fellowship that may prolong time to final career placement and stability. Altogether, many oncology trainees choose to delay pregnancy for career and social support reasons, and as such there is an increasing interest in fertility preservation.^9^ Assisted reproductive technology (ART) and its pricing have evolved to where they may be feasible and affordable for some women, and are becoming covered more often by insurance.^10,11,12^ However, attitudes among female oncologists regarding family planning are unknown. To that end, the objectives of this study were (1) to understand barriers to family planning in the context of one’s career, (2) to understand the association of fertility treatment options (such as ART) with career decisions, and (3) to assess experiences of pregnancy-based discrimination among female oncologists.

## Methods

A novel 39-item questionnaire was designed after comprehensive literature review to collect cross-sectional data on attitudes toward childbearing, fertility and family planning, ART, and discrimination (eAppendix in the Supplement). Input was gathered from female oncologists and fertility specialists and the survey was pilot-tested and assessed for face validity. The larger definition of family planning as “the ability of individuals and couples to anticipate and attain their desired number of children and the spacing and timing of their births … through use of contraceptive methods and the treatment of involuntary infertility” was used for the purposes of this survey.^13^ Data were collected anonymously while maintaining confidentiality via REDCap from May 7 to June 30, 2020. By answering this survey, participants responded that they agreed that they were adult female oncologists in the United States who could read and write English. The Memorial Sloan Kettering Cancer Center institutional review board granted exemption at the start of the study because data were deidentified. This study followed the American Association for Public Opinion Research (AAPOR) reporting guideline. The completion rate was defined as surveys with answers to all 39 questions divided by the number of participants who initiated the survey.

To ascertain the types of factors associated with family planning, “check all that apply” questions regarding positive and negative personal and professional factors as well as specific questions regarding fertility timing and practice environment were included. Questions regarding potential challenges to fertility, pregnancy, and maternity leave were asked, including about complications that occurred during pregnancy and use of ART. Detailed questions regarding support throughout training and in practice were also asked, as well as questions about discrimination of both a direct and an indirect nature. Opportunities to provide additional comments were available as an independent question and embedded throughout in several questions, which were categorized and organized for separate future qualitative analysis. Finally, demographic data were collected by self-report.

Survey links were distributed via email and/or social media channels (Twitter and Facebook) to members of the American College of Radiation Oncology, American Head and Neck Society, American Medical Women’s Association, American Society for Breast Surgeons, Association of Residents in Radiation Oncology, American Society for Clinical Oncology, American Society of Hematology, American Society of Pediatric Hematology/Oncology, American Society for Radiation Oncology, American Society for Transplantation and Cellular Therapy, Association of Women Surgeons, Hematology and Oncology Wolf Pack Group, Hematology & Oncology Women Physicians Group, Physician Moms Group, Radiation Oncology Women Physicians Group, Society of Gynecologic Oncology, Society of Surgical Oncology, Society of Urologic Oncology, Society for Women in Radiation Oncology, and Women Physician Warriors. The target group was US female oncologists of all career levels including surgical, gynecologic, pediatric, medical, and radiation oncologists. The range of respondents included medical students with plans for a career in oncology to retirees. The survey was open to adult female oncologists (including female-identifying women and those assigned female at birth) in the United States who could read and write English. Data were collected anonymously while maintaining confidentiality and descriptive statistics were generated. Workforce data were obtained from the Association of American Medical Colleges to calculate the percentage of respondents from each specialty. No compensation was provided for completing the survey and participant validity was obtained by asking a simple addition question prior to initiation of the survey.

Questions related to fertility, family planning, ART, and discrimination by field of oncology were assessed using χ^2^ analysis. Bonferroni correction was applied for pairwise comparisons. Multivariable logistic regression was performed to determine independent variables for discrimination experienced during maternity leave. Factors included race and ethnicity, field of oncology, age of first birth, number of children, and timing of first birth. All *P* values were 2-sided, with *P* < .05 representing significance. Statistical analyses were performed in SPSS, version 23 (SPSS Inc).

## Results

### Demographic Characteristics

Responses were collected from 1004 female oncologists, with a 96.9% completion rate (the percentage of those who completed the entire survey; 973 of 1004), comprised of 351 respondents (35.0%) practicing in radiation oncology, 344 (34.3%) in medical oncology, 185 (18.4%) in surgical or gynecologic oncology, and 91 (9.1%) in pediatric oncology. The response rate was unknown, as the reach of those who may have seen the survey could not be ascertained given the method of survey distribution via social media channels. The total number of participants may have represented approximately 8.1% of all known female medical oncologists, 13.5% of all known female pediatric oncologists, 23.1% of all known female radiation oncologists, and 58.2% of all known female surgical oncologists, including gynecologic oncologists, per the 2018 American Medical Association data report of the oncology workforce.^14^ The distribution of current age was as follows: 54 (5.4%) were 25 to 30 years, 263 (26.2%) were 31 to 35 years, 305 (30.4%) were 36 to 40 years, 206 (20.5%) were 41 to 45 years, 73 (7.3%) were 45 to 50 years, 49 (4.9%) were 51 to 55 years, 21 (2.1%) were 55 to 60 years, and 35 (3.5%) were older than 60 years. Most respondents (847 [84.4%]) were married and 713 (71.0%) were currently working full-time (Table 1). Of those who were in relationships, most (558 [55.6%]) had a partner in a field other than medicine, while 296 (29.5%) had a partner who is a physician. A total of 802 respondents (79.9%) were in full practice, while 194 (19.3%) were in training. Most (616 [61.4%]) were practicing at an academic medical center.

**Table 1.** Demographic Characteristics

Characteristic	No. (%) (N = 1004)
Marital status	
Married	847 (84.4)
Partner employment status	
Full-time	746 (74.3)
Partner in medicine	
My partner is not in medicine	558 (55.6)
My partner is in medicine but is not a physician (ie, pharmacy, nurse, physicist)	61 (6.1)
My partner is a physician	296 (29.5)
Sexual orientation	
Heterosexual	966 (96.2)
Level of training	
Attending physician	
>20 y After residency or fellowship	69 (6.9)
11-20 y After residency or fellowship	173 (17.2)
6-10 y After residency or fellowship	215 (21.4)
Within 5 y of residency or fellowship	345 (34.4)
Medical student on an oncology career path	2 (0.2)
Postgraduate trainee	192 (19.1)
Other (retired, unemployed, etc)	8 (0.8)
Practice setting	
Academic	616 (61.4)
Hospital-based or private practice	194 (19.3)
Work hours and salary payment model	
5 d/wk, Clinic or research time	713 (71.0)
Fixed salary	304 (30.3)
Base salary with productivity-based bonus	269 (26.8)
Productivity model	98 (9.8)
Race and ethnicity	
Asian	217 (21.6)
Black or African American	44 (4.4)
Hispanic or Latina	57 (5.7)
White	681 (67.8)
All other^a^	44 (4.4)
Do you have children?	
Yes, I gave birth	735 (73.2)
Yes, partner gave birth	6 (0.6)
Yes, stepchildren, surrogate, or adopted	22 (2.2)
No, I desire to	190 (18.9)
No, I do not plan to	34 (3.4)
No, I do not know if I want to	32 (3.2)
If you have children, at what age did you start trying to have children?	
21-25 y	17 (1.7)
26-30 y	267 (26.6)
31-35 y	358 (35.7)
36-40 y	96 (9.6)
41-45 y	8 (0.8)
NA (I do not have children or my pregnancy was accidental)	258 (25.7)
What age did you give birth to your first child?	
<25 y	16 (1.6)
26-30 y	202 (20.1)
31-35 y	384 (38.2)
36-40 y	128 (12.7)
41-45 y	13 (1.3)
NA (I do not have children or I did not carry the pregnancy)	261 (26.0)
When in your career did you give birth to your first child?	
Before medical school	14 (1.4)
During medical school	43 (4.3)
During residency	211 (21.0)
During fellowship (or other postdoctoral training)	204 (20.3)
As an attending physician	275 (27.4)
NA (I do not have children or I did not carry the pregnancy)	257 (25.6)
No. of children	
0	245 (24.4)
1	209 (20.8)
2	373 (37.2)
3	141 (14.0)
4	24 (2.4)
≥5	5 (0.5)

Abbreviation: NA, not applicable.

^a^ Included those who identified as Middle Eastern and any other race and ethnicity that was not represented in the choices provided.

Most respondents (763 [76.0%]) had children, and of those, 625 (81.9%) started trying to have a child between 26 and 35 years of age, and 586 (76.8%) reported giving birth to their first child between 26 and 35 years of age, with an additional 141 (18.5%) reported having their first child between 36 and 45 years of age (Table 1). A total of 415 women (41.3%) first gave birth during postgraduate training, while 275 (27.4%) first gave birth in years 1 to 5 as an attending physician. See Table 1 for full demographic and family planning details on the sample.

### Factors Associated With Family Planning

About one-fourth of female physicians (243 [24.2%]) said that family planning was associated with their career decision of academics vs private practice, and 951 (94.7%) stated that their career plans were at least somewhat associated with the timing of when to start a family. The most commonly reported positive factors associated with fertility planning were a supportive partner (802 [79.9%]), income as an attending physician (379 [37.7%]), nearby family support (281 [28.0%]), having a female mentor or role model (241 [24.0%]), and having a supportive program during training (217 [21.6%]). The most commonly reported negative factors associated with fertility planning were long work hours and heavy workload (669 [66.6%]), concern for fertility or biological clock (478 [47.6%]), income as a trainee (406 [40.4%]), lack of nearby family support (372 [37.1%]), and bad or nonexistent maternity leave policy during training (314 [31.3%]) or as an attending physician (175 [17.4%]).

### Pregnancy Complications, Infertility, and ART

Of all respondents who had been pregnant, approximately two-thirds (511 of 778 [65.7%]) had some type of pregnancy complication. Approximately one-third of respondents (318 [31.7%]) reported having experienced a miscarriage after a confirmed pregnancy; of those, most (196 [61.6%]) reported 1 miscarriage, while the remainder had 2 or more miscarriages (122 [38.4%]) (Table 2). Approximately one-third of respondents (315 [31.4%]) reported difficulty with infertility that required fertility counseling and/or treatment. Among those who had fertility concerns, 89 of 501 (17.8%) had mental health concerns owing to infertility diagnosis and/or treatments.

**Table 2.** Fertility, Family Planning, and ART by Field of Oncology

Characteristic	Oncologists, No. (%)						χ^2^ (No.)	*df*	*P* value	SS pairwise comparisons
	All (N = 1004)	Med onc (n = 344)	Other (n = 32)	Ped onc (n = 91)	Rad onc (n = 351)	Surg onc (n = 186)				
Was your family planning associated with your career decision re: academics vs private practice?										
No	629 (62.6)	206 (59.9)	24 (77.4)	77 (84.6)	185 (52.7)	135 (73.0)	57.0 (1004)	15	<.001	Med onc vs ped onc; med onc vs surg onc; ped onc vs rad onc; rad onc vs surg onc
Yes	243 (24.2)	99 (28.8)	3 (9.7)	10 (11.0)	100 (28.5)	31 (16.8)				Med onc vs ped onc; med onc vs surg onc; ped onc vs rad onc; rad onc vs surg onc
Unsure	126 (12.5)	39 (11.3)	4 (12.9)	3 (3.3)	62 (17.7)	18 (9.7)				Ped onc vs rad onc
No. of miscarriages after confirmed pregnancy										
0	457 (45.5)	172 (50.0)	8 (25.8)	41 (45.1)	156 (44.4)	80 (43.2)	40.3 (1004)	25	.03	None
1	196 (19.5)	66 (19.2)	10 (32.3)	20 (22.0)	58 (16.5)	42 (22.7)				None
2	75 (7.5)	27 (7.8)	4 (12.9)	10 (11.0)	16 (4.6)	17 (9.2)				None
≥3	47 (4.7)	14 (4.1)	2 (6.5)	3 (3.3)	16 (4.6)	12 (6.5)				None
NA (I have never been pregnant)	225 (22.4)	63 (18.3)	7 (22.6)	17 (18.7)	103 (28.3)	34 (18.4)				Med onc vs rad onc
Difficulties with infertility or required fertility counseling and/or treatment										
No	493 (49.1)	179 (52.0)	10 (32.3)	40 (44.0)	171 (48.7)	92 (49.7)	41.3 (1004)	20	.003	None
Yes	315 (31.4)	117 (34.0)	14 (45.2)	40 (44.0)	85 (24.2)	59 (31.9)				Ped onc vs rad onc
Unsure	16 (1.6)	6 (1.7)	0	2 (2.2)	5 (1.4)	3 (1.6)				None
NA (I have not attempted to have children)	177 (17.6)	41 (11.9)	7 (22.6)	9 (9.9)	89 (25.4)	30 (16.2)				Med onc vs rad onc; ped onc vs rad onc

Abbreviations: ART, assisted reproductive technology; med onc, medical oncology; NA, not applicable; ped onc, pediatric oncology; rad onc, radiation oncology; SS, statistically significant; surg onc, surgical oncology.

On the topic of ART, 164 participants (16.3%) reported the use of fertility medications, and 53 (5.3%) reported cryopreservation of eggs. A total of 130 respondents (12.9%) reported the use of intrauterine insemination and 133 (13.2%) reported the use of in vivo fertilization. Among those who experienced fertility concerns, 36.6% (232 of 634) reported facing financial burden because of fertility or pregnancy that was in some way associated with their career choice. When asked if fertility preservation should be discussed with women during medical school and/or residency, 660 respondents (65.7%) stated it should.

### Discrimination

One-third of respondents (312 [31.1%]) reported not having support during training for issues associated with fertility and/or pregnancy, while 180 (17.9%) stated that they were explicitly told to return to work before maternity leave was over or that it was implied that they should return before maternity leave was over (Table 3). Among the specialties, surgical oncologists experienced this sentiment the most (39 of 186 [21.0%]), while pediatric oncologists reported this the least (15 of 91 [16.5%]; *P* = .004). One third of all respondents (322 [32.1%]) stated they had experienced either subtle or overt discrimination for being pregnant, while 332 (33.1%) stated they experienced discrimination for taking maternity leave. There were significant differences in discrimination rates noted during pregnancy, with the lowest reported among pediatric oncologists at (18 of 91 [19.8%]; *P* < .001), while up to 33% of all other specialties experienced discrimination.

**Table 3.** Discrimination and Support With Department by Field of Oncology

Characteristic	Oncologists, No. (%)						χ^2^ (No.)	*df*	*P* value	SS pairwise comparisons
	All (N = 1004)	Med onc (n = 344)	Other (n = 32)	Ped onc (n = 91)	Rad onc (n = 351)	Surg onc (n = 186)				
Support at current workplace during training for issues associated with fertility and/or pregnancy?										
No	312 (31.1)	117 (34.0)	9 (29.0)	18 (19.8)	116 (33.0)	51 (27.6)	23.3 (1004)	20	.27	None
Yes	324 (32.3)	118 (34.3)	8 (25.8)	30 (33.0)	104 (29.6)	64 (34.6)				None
Unsure	128 (12.7)	36 (10.5)	3 (9.7)	19 (20.9)	47 (13.4)	22 (11.9)				None
NA (I did not consider fertility or pregnancy during training)	237 (23.6)	72 (20.9)	11 (35.5)	24 (26.4)	82 (23.4)	48 (25.9)				None
Were you ever encouraged to take less maternity leave than was policy for your institution?										
No	522 (52.0)	197 (57.3)	12 (38.7)	55 (60.4)	160 (45.6)	98 (53.0)	47.7 (1004)	25	.004	Med onc vs rad onc
Yes, told that I should return to work before maternity leave was over	53 (5.3)	22 (6.4)	1 (3.2)	5 (5.5)	12 (3.4)	13 (7.0)				None
Yes, it was implied	127 (12.6)	44 (12.8)	6 (19.4)	10 (11.0)	41 (11.7)	26 (14.1)				None
Unsure	45 (4.5)	11 (3.2)	3 (9.7)	2 (2.2)	19 (5.4)	9 (4.9)				None
NA (I have never been pregnant or had a child before I joined the institution)	252 (25.1)	67 (19.5)	9 (29.0)	19 (20.9)	117 (33.3)	39 (21.1)				Med onc vs rad onc; rad onc vs surg onc
Did you ever feel discriminated (either subtle or overt) against for being pregnant?										
No	382 (38.0)	140 (40.7)	7 (22.6)	47 (51.6)	108 (30.8)	79 (42.7)	63.9 (1004)	30	<.001	Ped onc vs rad onc
Yes, by both male and female colleagues	195 (19.4)	82 (23.8)	7 (22.6)	5 (5.5)	72 (20.5)	29 (15.7)				Med onc vs ped onc; ped onc vs rad onc
Yes, by male colleagues	106 (10.6)	22 (6.4)	6 (19.4)	11 (12.1)	39 (11.1)	28 (15.1)				Med onc vs surg onc
Yes, by female colleagues	21 (2.1)	10 (2.9)	0 (0)	2 (2.2)	5 (1.4)	4 (2.2)				None
Unsure	61 (6.1)	22 (6.4)	3 (9.7)	8 (8.8)	16 (4.6)	12 (6.5)				None
NA (I have never been pregnant)	234 (23.3)	67 (19.5)	8 (25.8)	18 (19.8)	107 (30.5)	33 (17.8)				Med onc vs rad onc; rad onc vs surg onc
Did you ever feel discriminated (either subtle or overt) against for taking maternity leave?										
No	375 (37.4)	136 (39.5)	12 (38.7)	42 (46.2)	113 (32.2)	72 (38.9)	70.8 (1004)	30	<.001	None
Yes, by both male and female colleagues	195 (19.4)	84 (24.4)	3 (9.7)	10 (11.0)	68 (19.4)	30 (16.2)				None
Yes, by male colleagues	95 (9.5)	24 (7.0)	6 (19.4)	7 (7.7)	35 (10.0)	23 (12.4)				None
Yes, by female colleagues	42 (4.2)	13 (3.8)	0	9 (9.9)	6 (1.7)	14 (7.6)				Ped onc vs rad onc; rad onc vs surg onc
Unsure	52 (5.2)	20 (5.8)	1 (3.2)	6 (6.6)	14 (4.0)	10 (5.4)				None
NA (I have never been pregnant)	240 (23.9)	66 (19.2)	9 (29.0)	17 (18.7)	112 (31.9)	35 (18.9)				Med onc vs rad onc; rad onc vs surg onc

Abbreviations: med onc, medical oncology; NA, not applicable; ped onc, pediatric oncology; rad onc, radiation oncology; SS, statistically significant; surg onc, surgical oncology.

On multivariable logistic regression, having more than 1 child (2 children: odds ratio [OR], 1.62; 95% CI, 1.10-2.39; *P* = .02; ≥3 children: OR, 1.84; 95% CI, 1.14-2.95; *P* = .01) was associated with increased likelihood of experiencing discrimination during maternity leave (Table 4). Race and ethnicity, field of oncology, maternal age at first birth, and career timing of first birth were not significantly associated with increased likelihood of discrimination during maternity leave.

**Table 4.** Multivariable Logistic Regression for Discrimination Experienced During Maternity Leave

Characteristic	OR (95% CI)	*P* value
Race and ethnicity		
Asian	0.99 (0.67-1.48)	.98
Black or African American	1.87 (0.75-4.66)	.18
Hispanic or Latina	0.94 (0.48-1.84)	.86
White	1 [Reference]	NA
Other	2.12 (0.87-5.15)	.10
Field of oncology		
Radiation oncology	1 [Reference]	NA
Surgery (including gynecology)	0.94 (0.60-1.49)	.79
Medical (including neurology)	0.90 (0.61-1.31)	.57
Pediatrics	0.64 (0.36-1.14)	.13
Age at first birth, y		
<30	1 [Reference]	NA
31-35	1.25 (0.82-1.89)	.30
>35	0.98 (0.53-1.80)	.95
No. of children		
1	1 [Reference]	NA
2	1.62 (1.10-2.39)	.02
≥3	1.84 (1.14-2.95)	.01
When in career gave birth to first child?		
Before residency	1 [Reference]	NA
During residency or fellowship	1.21 (0.65-2.26)	.54
During years as attending physician	1.42 (0.69-2.92)	.35

Abbreviations: NA, not applicable; OR, odds ratio.

## Discussion

In this large cross-sectional survey study of more than 1000 female oncologists, 1 in 3 respondents reported experiencing infertility, and more than 90% stated that their career influenced family planning. One-third reported experiencing discrimination during pregnancy and for taking maternity leave. These results add to the increasing number of studies showing barriers to success for female physicians that need to be addressed to achieve equity in the medical profession.

Although women now make up half of today’s medical students and trainees, they only comprise 37% of full-time academic physicians and 13% of full professors.^15^ Numerous studies have shown that demands associated with child-rearing play a significant role in female physicians’ careers, as personal and professional interests are often competing.^3,6,16,17^ Women feel they must delay having children for professional advancement^8^ or sacrifice their career to focus on motherhood. The most common negative factor associated with family planning in the present study was long work hours and heavy workload (66.6%), which was consistent with a 2016 study by Stentz et al,^1^ who found that 83% of women physicians were delaying pregnancy because of competing career demands. A more recent survey study of 327 women physician leaders also found the most common reason for delaying having children were demands of training (72.9%) and long work hours (61.5%).^17^ Systemic changes are needed by implementing policies at the national and institutional level for paid parental leave, offering subsidies for childcare, and creating avenues for equitable promotion of women in the field. Culturally, institutions can do better by increasing sensitivity training for gender-based discrimination as well as minimizing micro barriers such as rescheduling meeting times to typical work hours instead of times where a colleague may be taking care of children at home. Increasingly, women may also benefit from ART to improve opportunities to build a family, yet challenges persist, as 36.6% of respondents reported facing a financial burden with ART because of fertility or pregnancy that was in some way associated with their career choice.

The Pregnancy Discrimination Act was enacted in 1978 and prohibited sex discrimination on the basis of pregnancy. In the present study, discrimination faced by female oncologists during pregnancy and for taking maternity leave is a significant finding. One in 5 surgical oncologists reported implicit or explicit pressure to return to work before maternity leave was over. This finding may explain prior work showing that women surgeons were more likely to have no biological children (45%) than men in the same field.^18^

One-third of respondents reported the absence of maternity leave during training, and 17.9% reported the absence of maternity leave as an attending physician to be negative factors associated with family planning. These numbers are slightly better but consistent with 2 survey studies finding more than one-third of women cited no institutional policies with respect to time off after birth.^17,19^ One of these studies found that 10% of women reported working more during pregnancy, possibly to “make up time” from maternity leave.^17^ These findings suggest that even with institutional policies in place, there is still considerable work to be done with respect to fostering a supportive culture.

A recent study of 667 US female oncologists found that 21.9% considered leaving academia in the next 5 years given the gender inequalities, specifically associated with promotion and a sense of belonging in their work environment.^20^ This concern is likely similar in our sample of oncologists who face discrimination for fertility concerns; these sentiments likely start early, as a survey of radiation oncology trainees found that female residents were more responsible for childcare duties than their male counterparts but still maintained similar research productivity and career aspirations.^21^ Implementation of paid paternity leave across institutions would help achieve gender parity, as shown in Sweden, where such policies have allowed a more equitable division of labor in shared parenthood.^22^

The culture of gender inequity persists in various forms and at the national level. One study of oncologists identified by the American Society of Clinical Oncology Research Survey Pool found a significantly higher rate of sexual harassment experienced by cisgender women compared with their cisgender male counterparts (80% vs 56%; *P* < .001), which was adversely associated with mental health, sense of workplace safety, job satisfaction, and intentions to leave.^23^ Another study of speaker introductions at the American Society of Clinical Oncology Annual Meetings found that female speakers were more likely to be introduced by their first name only compared with their male peers.^24^ These studies underscore the challenges, whether implicit or explicit, that women may face at all points in their career that lead to burnout and ultimately an exodus of women from medicine. Our study adds to this literature by clarifying the association of fertility concerns with career decisions and highlighting the potentially toxic effects of discrimination against new mothers. Keenan and colleagues^25^ offer pragmatic solutions to achieve gender parity in the academic oncology workforce, including flexible work schedules, an equity-based approach to protected research time, and protection of staff from administrative burdens.

Until recently, there has been a dearth of policy at the programmatic, institutional, and national level allowing time and protection for pregnancy and maternity leave for trainees despite multiple calls for action.^3,26^ A significant development was made in July 2020 with the announcement by the American Board of Medical Specialties^27^ announcing the adoption of a progressive leave policy for residents and fellows allowing a minimum of 6 weeks away without exhausting time allowed for vacation or sick leave and without requiring an extension in training. Soon after a survey study showed that women radiation oncologists faced significant family planning hurdles as a result of limited examination offerings,^28^ the American Board of Radiology followed suit by increasing the flexibility of examination schedules and defining the maximal amount of time an individual can be absent from training and still graduate on time. Specifically, there has been broad support for 35 workdays per year averaged over a 4-year residency training in addition to 12 weeks for parental, caregiver, and medical, leave per American Board of Medical Specialties policy,^29^ although implementation is still institution dependent. For those in full practice, the Family and Medical Leave Act protects up to 12 weeks of unpaid maternity or family leave. There remains no national policy regarding paid parental leave, with variable policies at the state or employer level. Some working mothers ultimately depend on short-term disability to help cover their income losses during leave.^30^

Assisted reproductive technology should not be considered the solution to pregnancy discrimination; however, 66% of respondents stated that fertility preservation should be discussed during medical school and/or residency, as it can become an option for those whose family-building plans are delayed. The lack of education and structured policy requiring health insurance to cover ART benefits can exacerbate the emotional, physical, and financial effects of infertility. It has been shown that infertility, high-risk pregnancies, and miscarriages have been associated with higher rates of burnout^6^ and that physicians with more children and less home stress are significantly more likely to be satisfied.^31^

### Limitations

Although we had a large number of responses with a near 100% completion rate, there were several important limitations. First, our survey was novel to this study as, to our knowledge, there are no validated measures to assess fertility concerns in physicians. In addition, accurate response rates could not be calculated based on online recruitment tactics, as it was difficult to ascertain the reach of those who may have seen the survey through social media platforms. Selection bias is also a potential concern, as we may be targeting a more active group of participants who use social media and would engage in survey participation; thus, the results may not necessarily be representative of all women in oncology. Although estimates based on available Association of American Medical Colleges data show relatively high participation rates within each oncology specialty, the accuracy of the Association of American Medical Colleges data could not be validated so it is likely that the views reflect less than half of female oncologists in the US. Given the sensitive nature of the questions, participants may have answered what they think are socially acceptable responses; thus, there may be response bias in this regard. As circumstances associated with pregnancy and family planning could have occurred decades prior, there may additionally be recall bias in some cases. We also did not ask questions assessing the use of donor sperm to conceive either because of being single or in a same-sex relationship or questions about use of in vitro fertilization due to the need for genetic testing and not for infertility; thus, the survey did not capture the full extent of ART practices. Finally, all possible scenarios were not captured in the survey questions thus, the best answer choice instead of the most accurate may have been chosen.

## Conclusions

In the largest study to date, to our knowledge, of female physicians on the topic, a considerable proportion of women in oncology reported facing infertility and difficult career choices associated with family planning. Systemic changes are necessary early in medical education and training to ensure that women are supported and able to advance equitably in the field. Widespread implementation of paid pregnancy leave and provision of affordable ART are necessary steps in the right direction.

## Notes

Supplement. **eAppendix.** Family Planning, Fertility, and Career Decisions Questionnaire
