# Supplementary material for: Family Planning, Fertility, and Career Decisions Among Female Oncologists
Source: JAMA Netw Open. 2022 Oct 31;5(10):e2237558. doi: 10.1001/jamanetworkopen.2022.37558 (PMC9623435; doi:10.1001/jamanetworkopen.2022.37558)
Supplement: Supplement. — eAppendix. Family Planning, Fertility, and Career Decisions Questionnaire [file jamanetwopen-e2237558-s001.pdf]

Supplementary Online Content

Lee A, Kuczmarska-Haas A, Dalwadi SM, et al. Family planning, fertility, and career decisions among female oncologists. *JAMA Netw Open.* 2022;5(10):e2237558. doi:10.1001/jamanetworkopen.2022.37558

eAppendix. Family Planning, Fertility, and Career Decisions Questionnaire

This supplementary material has been provided by the authors to give readers additional information about their work.

© 2022 Lee A et al. *JAMA Network Open.*

eAppendix. Family Planning, Fertility, and Career Decisions Questionnaire

Family Planning, Fertility, and Career Decisions in Female Oncologists

Female oncologists spend their 20s and early 30s in training and establishing careers. Building a family during training or early career may be challenging but waiting may result in fertility concerns. Assisted reproductive technology (ART) and pricing is finally at the point where it may be feasible and affordable for some women however attitude and utilization for female oncologists is unknown.

This survey was designed as a clinical research project to assess possible barriers to family planning and to establish current use of ART. Responses are anonymous. Some questions are quite personal in nature; if you do not feel comfortable answering a question, please skip that question. Please email questions or concerns to PI Fumiko Chino, MD (chinof@mskcc.org) or call 848-225-6565.

This survey has 39 questions and will take 5-10 minutes to complete.

By answering this survey, I agree that I am an adult female* oncologist in the United States who can read and write English.

*including female identifying, assigned female at birth

1. Which of the following positive factors have influenced your decision on when to start a family? (check all that apply)

Positive: Supportive partner
Positive: Partner willing to be stay-at-home parent
Positive: School/residency/fellowship maternity leave policy
Positive: Job maternity leave policy
Positive: Nearby family support
Positive: Supportive program director/program (during training)
Positive: Supportive chair/program (as an attending)
Positive: Supportive colleagues (as an attending)
Positive: Income (as an attending)
Positive: Female mentor/role model
None of the above
Other________(free text)

Other

2. Which of the following negative factors have influence your decision on when to start a family? (check all that apply)

Negative: Lack of a partner
Negative: Lack of a supportive partner
Negative: Bad or non-existent school/residency/fellowship maternity leave policy
Negative: Bad or non-existent job maternity leave policy
Negative: Lack of nearby family support
Negative: Lack of support from program director/program (during training)
Negative: Lack of support of chair/program (as an attending)
Negative: Lack of support of colleagues (as an attending)
Negative: Long work hours/heavy workload
Negative: Income (as a resident/fellow)
Negative: Concern for fertility/"biological clock"
None of the above
Other________(free text)

Other

© 2022 Lee A et al. *JAMA Network Open.*

3. How much have your education/career plans influenced your decision of timing of when (or if) to start a family?

- ◯ Extremely influenced
- ◯ Very much influenced
- ◯ Moderately influenced
- ◯ Somewhat influenced
- ◯ Not influenced

4. Did your family planning influence your career decision re: academics versus private practice?

- ◯ Yes
- ◯ Unsure
- ◯ No

5. Do you have children? (check all that apply)

- Yes, I gave birth
- Yes, my partner gave birth
- Yes, I have stepchildren
- Yes, I used a surrogate
- Yes, I adopted
- No, but I desire to
- No, and I do not plan to have children
- No, and I don't know if I want children

6. If you have children, at what age did you start trying to have children?

- ◯ N/A (I do not have children)
- ◯ N/A (My pregnancy was accidental)
- ◯ < 20
- ◯ 21-25
- ◯ 26-30
- ◯ 31-35
- ◯ 36-40
- ◯ 41-45
- ◯ 46-50
- ◯ 51-55
- ◯ >55

7. If you have children, at what age did you give birth to your first child?

- ◯ N/A (I do not have children)
- ◯ N/A (I have children but I did not carry the pregnancy)
- ◯ < 20
- ◯ 21-25
- ◯ 26-30
- ◯ 31-35
- ◯ 36-40
- ◯ 41-45
- ◯ 46-50
- ◯ 51-55
- ◯ >55

8. If you have children, when in your career did you have your first child?

- ◯ N/A (I do not have children)
- ◯ N/A (I have children but I did not carry the pregnancy)
- ◯ Before college
- ◯ During college
- ◯ After college but before medical school
- ◯ During medical school
- ◯ During residency
- ◯ During fellowship (or other post doctoral training)
- ◯ In year 1-5 as an attending
- ◯ After year 5 as an attending

© 2022 Lee A et al. *JAMA Network Open.*

9. How many children do you have?

- ◯ 0
- ◯ 1
- ◯ 2
- ◯ 3
- ◯ 4
- ◯ 5 or more

10. How many miscarriages after confirmed pregnancies have you had?

- ◯ N/A (I have never been pregnant)
- ◯ 0
- ◯ 1
- ◯ 2
- ◯ 3 or more

11. Have you had difficulties with infertility or required fertility counseling and/or treatment?

- ◯ N/A (I have not attempted to have children)
- ◯ Yes
- ◯ Unsure
- ◯ No

12. Have you had any pregnancy and/or delivery complications? (check all that apply)

N/A (I have never been pregnant)
No
Multiple pregnancy (twins, triplets, etc)
Gestational diabetes
Hyperemesis gravidarum
High blood pressure
Preeclampsia
Premature labor and/or premature rupture of membranes
Failture to progress
Cervical insufficiency
Fetal malformation
Chromosomal abnormalities
Fetal malposition
Chorioamnionitis
Emergent/unplanned C-section
Miscarriage (< 20 weeks)
Fetal demise (>20 weeks)
Mental health concerns
1-5 days missed from work due to complications
More than a week missed from work due to complications
Other________(free text)

Other

13. Have you used any of the following assisted reproductive technologies? (check all that apply)

N/A (I have not attempted to have children)
No
Fertility medications (ex: Clomid)
Cryopreservation
Intrauterine insemination (IUI)
In vitro fertilization (IVF)
Surrogacy (using my own eggs to have a biological child)
Surrogacy (used donor eggs for a non-biological child)
Other________(free text)

Other

© 2022 Lee A et al. *JAMA Network Open.*

14. What out-of-pocket costs have you incurred due to fertility concerns (in your attempts to have a child)?

- ◯ N/A (I have not attempted to have children or did not need fertility treatment)
- ◯ $0
- ◯ $1-499
- ◯ $500-999
- ◯ $1,000-4,999
- ◯ $5,000-$9,999
- ◯ $10,000-19,999
- ◯ $20,000-29,999
- ◯ $30,000 or above

15. Have you faced financial burden due to fertility or pregnancy which you feel is unique to your career choice?

- ◯ N/A (I have not experienced fertility concerns)
- ◯ Yes, because I am a female doctor
- ◯ Yes, primarily because I am a female oncologist (i.e. other female doctors wouldn't necessarily have this burden)
- ◯ Yes, because I am both a female doctor and a female oncologist
- ◯ Unsure
- ◯ No

16. Have you faced any adverse effects from fertility diagnosis and/or treatments? (check all that apply)

N/A (I have not experienced fertility concerns)
No
Yes, ovarian hyperstimulation syndrome
Yes, mental health concerns
Yes, 1-5 days missed from work due to symptoms
Yes, more than a week missed from work due to symptoms
Other________(free text)

Other

© 2022 Lee A et al. *JAMA Network Open.*

17. Fertility preservation is the freezing of eggs or embryos for possible use in the future. Which of the statements below most closely aligns with your feelings about this technology? [Training is considered: college, medical school, residency, fellowship]

◯ I had children during training and I do not feel that fertility preservation would have benefited me.
◯ I had children during training but I feel that fertility preservation would have benefited me (i.e. would have considered it as an option if it had been affordable/available).
◯ I did not have children during training but when I wanted to get pregnant, I did. I do not feel that fertility preservation would have benefited me.
◯ I did not have children during training and when I wanted to have children, I struggled to get pregnant. I feel that fertility preservation would have benefited me (i.e. would have considered it as an option if it had been affordable/available).
◯ I have used fertility preservation during training or as an attending.
◯ I am planning on using fertility preservation during training or as an attending if I can work out the logistics.
◯ I would like to pursue fertility preservation during training or as an attending, but I am not sure if this is feasible for me.
◯ I am conflicted about pursuing fertility preservation
◯ I don't have specific plans right now
◯ Other________(free text)

16 Other

18. Do you feel that fertility preservation should be specifically discussed with women during medical school and/or residency?

◯ Yes
◯ Unsure
◯ No

19. Did you feel supported by your workplace during training for issues related to fertility and/or pregnancy?

◯ N/A (I did not even consider fertility or pregnancy during training)
◯ Yes
◯ Unsure
◯ No

© 2022 Lee A et al. *JAMA Network Open.*

20. What is/was the maternity leave policy during residency/fellowship? (check all that apply)

No formal maternity leave policy, has to be negotiated
Formal maternity leave with < 4 weeks time off, more time needs to be negotiated
Formal maternity leave with 4 to 6 weeks time off, more time needs to be negotiated
Formal maternity leave with >6 but ≤8 weeks time off, more time needs to be negotiated
Formal maternity leave with >8 weeks time off
There is some dedicated paid time off (which is not vacation, sick time, or research time)
I had to use sick time
I had to use vacation time I
had to use research time
I had to use short term disability
I had to use FMLA (unpaid leave)
I took < 4 weeks
I took 4-6 weeks
I took >6 but ≤8 weeks
I took >8 weeks
I don't know/Unsure
Other________(free text)

Other

21. What is/was the maternity leave policy at your job? (check all that apply)

N/A (I am still in training)
No formal maternity leave policy, has to be negotiated
Formal maternity leave with < 4 weeks time off, more time needs to be negotiated
Formal maternity leave with 4 to 6 weeks time off, more time needs to be negotiated
Formal maternity leave with >6 but ≤8 weeks time off, more time needs to be negotiated
Formal maternity leave with >8 weeks time off
There is some dedicated paid time off (which is not vacation, sick time, or research time)
I had to use sick time
I had to use vacation time
I had to use short term disability
I had to use FMLA (unpaid leave)
I took < 4 weeks
I took 4-6 weeks
I took >6 but ≤8 weeks
I took >8 weeks
I don't know/Unsure
Other________(free text)

Other

22. Were you ever encouraged to take less maternity leave than was policy for your institution?

◯ N/A (I have never been pregnant or had a child before I joined the institution)
◯ Yes, someone told me that I should return to work before my maternity leave was over
◯ Yes, it was implied that I should return to work before my maternity leave was over
◯ Unsure
◯ No

© 2022 Lee A et al. *JAMA Network Open.*

23. Did you ever feel discriminated (either subtle or overt) against for being pregnant?

◯ N/A (I have never been pregnant)
◯ Yes, by both male and female colleagues
◯ Yes, by male colleagues alone
◯ Yes, by female colleagues alone
◯ Unsure
◯ No

24. Did you ever feel discriminated (either subtle or overt) against for taking maternity leave?

◯ N/A (I have never been pregnant)
◯ Yes, by both male and female colleagues
◯ Yes, by male colleagues alone
◯ Yes, by female colleagues
◯ Unsure
◯ No

25. Were you offered non-radiation exposing (ie. no brachytherapy or intraoperative fluoroscopy) assignments during pregnancy?

◯ N/A (I have never been pregnant)
◯ N/A (I do not do anything involving possible radiation exposure in my practice)
◯ Yes, I was automatically reassigned or someone else performed that aspect of procedures
◯ Yes, but I had to ask them
◯ Yes, but I wanted to continue to do procedures
◯ No, but it wasn't pertinent to my job at that time
◯ No

26. Do you have any other comments?________(free text)

27. How do you identify?

◯ Female
◯ Male
◯ Non-binary or other non-cis gender identity
◯ Other________(free text)

Other

28. What is your current age?

◯ < 25
◯ 25-30
◯ 31-35
◯ 36-40
◯ 41-45
◯ 46-50
◯ 51-55
◯ 56-60
◯ > 60

29. What is your marital status? (check all that apply)

Single
Married
Divorced
Widowed
In a relationship
Separated

30. If you have a partner, do they work? (check all that apply)

N/A (I do not have a partner)
They work full time
They work part time
They are able to work from home
They are retired
They are disabled

© 2022 Lee A et al. *JAMA Network Open.*

31. If you have a partner, are they also in medicine?

- ◯ N/A (I do not have a partner)
- ◯ My partner is not in medicine
- ◯ My partner is in medicine but is not a physician (i.e. pharmacy, nurse, physicist)
- ◯ My partner is a physician

32. What is your sexual orientation?

- ◯ Heterosexual
- ◯ Homosexual
- ◯ Bisexual
- ◯ Pansexual
- ◯ Asexual
- ◯ Other________(free text)

Other

33. What is your current level of training or career status?

- ◯ Medical Student on an Oncology career path
- ◯ PGY-1
- ◯ PGY-2
- ◯ PGY-3
- ◯ PGY-4
- ◯ PGY-5
- ◯ PGY-6
- ◯ PGY-7 or above
- ◯ Attending, within 5 years of residency or fellowship
- ◯ Attending, 6-10 years after residency or fellowship
- ◯ Attending, 11-15 years after residency or fellowship
- ◯ Attending, 16-20 years after residency or fellowship
- ◯ Attending, > 20 years after residency or fellowship
- ◯ Unemployed
- ◯ On disability
- ◯ Retired

34. What is your field of oncology?

- ◯ Radiation Oncology
- ◯ Medical Oncology (including Neuro-oncology)
- ◯ Surgical Oncology (including Gynecological Oncology)
- ◯ Pediatric Oncology
- ◯ Other________(free text)

Other

35. Where do you practice oncology?

- ◯ N/A (I am still in training)
- ◯ Academic medical center, basic science research track
- ◯ Academic medical center, clinical research track
- ◯ Academic medical center, clinical focus
- ◯ Veteran affairs (VA)
- ◯ Hospital employee, non-academic
- ◯ Private Practice
- ◯ Private Industry (including Pharmaceutical)
- ◯ Other_______(free text)

Other

© 2022 Lee A et al. *JAMA Network Open.*

36. What are your work hours and salary payment model? (check all that apply)

N/A (not currently working due to personal decision, disability, etc.)
Full time (5 days a week, clinic or research time)
Part time (< 5 days a week)
Fixed salary
Base salary with productivity-based bonus
Productivity model, with individual goals
Productivity mode, with group goals
Other________(free text)

Other

37. What is your race/ethnicity? (check all that apply)

White
Black/African American
Asian
American Indian or Alaska Native
Native Hawaiian or other Pacific Islander
Hispanic or Latina
Middle Eastern
Other________(free text)

Other

38. What is your religion?

◯ Christian/Protestant
◯ Christian/Catholic
◯ Christian/other
◯ Jewish (Orthodox)
◯ Jewish (Non-Orthodox)
◯ Hindu
◯ Muslim
◯ Buddhist/Taoist
◯ Atheist
◯ Agnostic
◯ Spiritual
◯ Other________(free text)

Other

39. How did you hear about this survey? (check all that apply)

Hematology & Oncology Wolf Pack Facebook Group
Radiation Oncology Women Physicians Facebook Group
Society for Women in Radiation Oncology
From a fellow female oncologist
Twitter
Emailed to me directly from study team
Other________(free text)

Other

© 2022 Lee A et al. *JAMA Network Open.*
